# Impact of low‐level viraemia on virological failure among Asian children with perinatally acquired HIV on first‐line combination antiretroviral treatment: a multicentre, retrospective cohort study

**DOI:** 10.1002/jia2.25550

**Published:** 2020-07-06

**Authors:** Tavitiya Sudjaritruk, Sirinya Teeraananchai, Azar Kariminia, Keswadee Lapphra, Nagalingeswaran Kumarasamy, Moy S Fong, Rawiwan Hansudewechakul, Torsak Bunupuradah, Penh Sun Ly, Revathy A Nallusamy, Annette H Sohn, Virat Sirisanthana, TREAT Asia Pediatric HIV Observational Database of IeDEA Asia‐Pacific, J Tucker, N Kumarasamy, C Ezhilarasi, A Kinikar, V Mave, S Nimkar, N Kurniati, D Muktiarti, SM Fong, M Lim, F Daut, P Mohamad, TJ Mohamed, R Nallusamy, T Sudjaritruk, V Sirisanthana, L Aurpibul, P Ounchanum, R Hansudewechakul, S Denjanta, A Kongphonoi, P Lumbiganon, P Kosalaraksa, P Tharnprisan, T Udomphanit, G Jourdain, T Puthanakit, S Anugulruengkit, W Jantarabenjakul, R Nadsasarn, K Chokephaibulkit, K Lapphra, W Phongsamart, S Sricharoenchai, KH Truong, QT Du, CH Nguyen, VC Do, TM Ha, LV Nguyen, DTK Khu, LT Nguyen, ON Le, AH Sohn, JL Ross, T Suwanlerk, MG Law, A Kariminia

**Affiliations:** ^1^ Department of Pediatrics, Faculty of Medicine Chiang Mai University Chiang Mai Thailand; ^2^ Research Institute for Health Sciences Chiang Mai University Chiang Mai Thailand; ^3^ The HIV Netherlands Australia Thailand Research Collaboration The Thai Red Cross AIDS Research Centre Bangkok Thailand; ^4^ Department of Statistics, Faculty of Science Kasetsart University Bangkok Thailand; ^5^ The Kirby Institute UNSW Australia Sydney Australia; ^6^ Department of Pediatrics, Faculty of Medicine Siriraj Hospital Mahidol University Bangkok Thailand; ^7^ Chennai Antiviral Research and Treatment Clinical Research Site VHS‐Infectious Diseases Medical Centre VHS Chennai India; ^8^ Hospital Likas Kota Kinabalu Malaysia; ^9^ Chiangrai Prachanukroh Hospital Chiang Rai Thailand; ^10^ National Centre for HIV/AIDS, Dermatology and STDs Phnom Penh Cambodia; ^11^ Penang Hospital Penang Malaysia; ^12^ TREAT Asia/amfAR – The Foundation for AIDS Research Bangkok Thailand

**Keywords:** viraemia, viral blip, viral rebound, paediatric, treatment failure, Asia

## Abstract

**Introduction:**

The clinical relevance of low‐level viraemia (LLV) and virological outcomes among children living with HIV (CLHIV) remains controversial. This study aimed to determine the impact of LLV on virological failure (VF) among Asian CLHIV on first‐line combination antiretroviral therapy (cART).

**Methods:**

CLHIV aged <18 years, who were on first‐line cART for ≥12 months, and had virological suppression (two consecutive plasma viral load [pVL] <50 copies/mL) were included. Those who started treatment with mono/dual antiretroviral therapy, had a history of treatment interruption >14 days, or received treatment and care at sites with a pVL lower limit of detection >50 copies/mL were excluded. LLV was defined as a pVL 50 to 1000 copies/mL, and VF as a single pVL >1000 copies/mL. Baseline was the time of the second pVL < 50 copies/mL. Cox proportional hazards models were performed to assess the association between LLV and VF.

**Results:**

From January 2008 to September 2016, 508 CLHIV (55% female) were eligible for the study. At baseline, the median age was 9.6 (IQR: 7.0 to 12.3) years, cART duration was 1.4 (IQR: 1.3 to 1.8) years, 97% of CLHIV were on non‐nucleoside reverse transcriptase inhibitor‐based regimens, and the median CD4 was 25% (IQR: 20% to 30%). Over a median follow‐up time of 6.0 (IQR: 3.1 to 8.9) years from baseline, 86 CLHIV (17%) had ever experienced LLV, of whom 32 (37%) had multiple LLV episodes. Female sex, living in Malaysia (compared to Cambodia), having family members other than biological parents/grandparents as a primary caregiver, and baseline CD4 < 25% increased risk of LLV. Overall, 115 children (23%) developed VF, corresponding to a rate of 4.0 (95%CI: 3.4 to 4.9) per 100 person‐years of follow‐up (PYFU). VF was greater among children who had ever experienced LLV compared with those who maintained virological suppression throughout the study period (8.9 vs. 3.3 per 100 PYFU; *p* < 0.001). In multivariable analyses, ever experiencing LLV was associated with increased risk of subsequent VF (adjusted hazard ratio: 3.01; 95%CI: 1.97 to 4.60).

**Conclusions:**

LLV increased the risk of subsequent VF among Asian CLHIV who had previously been suppressed on first‐line cART. Adherence interventions and additional targeted pVL monitoring may be warranted among children with LLV to facilitate early detection of VF.

## 1 INTRODUCTION

HIV plasma viral load (pVL) is an important tool to monitor treatment response and assess medication adherence in people living with HIV. One of the primary aims of combination antiretroviral therapy (cART) is to achieve and maintain pVL below the lower level of detection [1, 2, 3]. However, what is considered undetectable viral load varies by the technical properties of the laboratory assay used. Multiple international HIV treatment guidelines state that viral suppression at a pVL < 50 copies/mL is preferable as a hallmark of HIV treatment success and optimal long‐term clinical outcomes [2, 3].

During the course of cART, people living with HIV may experience small increases in pVL (pVL 50 to 1000 copies/mL) that do not reach the threshold for viral failure, known as low‐level viraemia (LLV). LLV can be transient, which is characterized by a brief increase in pVL followed by subsequent virological suppression, or persistent, which is a sustained increase in pVL over time. Transient LLV has been found in approximately 11% to 34% of adults living with HIV (ALHIV) in the United States, Canada, Spain and Switzerland on stable cART regimens [4, 5, 6, 7], and in 22% of children living with HIV (CLHIV) in the UK and Ireland on stable and previously suppressive cART [8]. Persistent LLV has been identified in between 9% and 56% of ALHIV in the United States, Canada and France [9, 10, 11]. The exact nature and aetiology of LLV are uncertain, but multifactorial causes have been proposed, including concomitant infections, vaccinations and low plasma drug concentrations due to suboptimal adherence to cART [12, 13, 14].

The clinical consequences of LLV among patients on previously suppressive cART have been controversial. Some previous studies among ALHIV have found no association between transient LLV and adverse virological and immunological outcomes [15, 16], whereas others have demonstrated a relationship between both transient and persistent LLV with subsequent virological failure (VF) [9, 10, 11, 17, 18, 19, 20, 21]. To date, the information regarding clinical outcomes of CLHIV experiencing LLV is limited, and the optimal clinical management for these individuals remains unclear. Furthermore, international paediatric guidelines provide limited recommendations around appropriate management [1, 2, 3]. The aim of this study was to assess the impact of LLV on virological outcomes in a cohort of Asian CLHIV who have been clinically stable and virologically suppressed on first‐line cART.

## 2 METHODS

### 2.1 Study cohort

The TREAT Asia Pediatric HIV Observational Database (TApHOD) is a multinational, multicentre, prospective observational cohort of CLHIV in the Asia‐Pacific region, established in 2008. TREAT Asia/amfAR (Bangkok, Thailand) is the study coordinating centre, and the Kirby Institute, University of New South Wales (Sydney, Australia) is the data management and statistical analysis centre for the cohort, which contributes to the International Epidemiology Databases to Evaluate AIDS (IeDEA) global consortium. The details of data collection have previously been published [22]. In September 2016, TApHOD included data from 5668 CLHIV submitted from 16 university‐based or public paediatric HIV sites in Cambodia (1 site; n = 754), India (1 site; n = 240), Indonesia (2 sites; n = 335), Malaysia (4 sites; n = 399), Thailand (5 sites; n = 2096) or Vietnam (3 sites; n = 1844). Ethics approval for this study was obtained from all participating sites, the study coordinating centre (TREAT Asia/amfAR), and the data management and statistical analysis centre (the Kirby Institute, University of New South Wales). Participant and/or parental consent as well as assent (if applicable) were deferred to each individual site and their institutional review board.

### 2.2 Study population

The study population consisted of Asian CLHIV followed in the cohort during January 2008 to September 2016. Eligible participants were CLHIV aged <18 years, and (1) who had perinatally acquired HIV infection; (2) whose first cART regimen was a combination of two nucleoside reverse transcriptase inhibitors (NRTI) with either a non‐nucleoside reverse transcriptase inhibitor (NNRTI) or a protease inhibitor‐boosted with ritonavir (PI/r), which was continued for ≥12 months and (3) who had a documented history of virological suppression, defined as having pVL < 50 copies/mL on two consecutive occasions over a period of at least six months after cART initiation. CLHIV who (1) were started treatment with mono or dual antiretroviral therapy, (2) had history of treatment interruption for >14 days or (3) received HIV treatment and care at sites with pVL lower level of detection >50 copies/mL were excluded from this study.

### 2.3 Data collection

Demographic and HIV‐related characteristics of eligible CLHIV were abstracted from the TApHOD database for this retrospective cohort analysis. Weight and height were based on the available recorded data closest to, but not more than 90 days before or 30 days after the date of interest. Weight (WAZ) and height for age z‐scores (HAZ) were calculated according to the 1977 National Center for Health Statistics/World Health Organization (WHO) reference [23], and the 2007 WHO growth references [24] respectively. CD4 T‐cell percentage and count at cART initiation were the nadir CD4 values documented closest to, but no more than, 180 days before or seven days after the date of cART initiation. pVL at cART initiation was the highest pVL level which children experienced closest to, but no more than, 180 days before or no later than the time of cART initiation. Other HIV‐related characteristics, including WHO clinical stage, immunological profiles, cART regimens and HIV disclosure status were based on the last available recorded information as of the date of interest (e.g. ART initiation).

### 2.4 Definition of low‐level viraemia

In this study, LLV was described as an at least one pVL level between 50 and 1000 copies/mL without a change of treatment regimen. This definition was applied because this level of viraemia would be considered an indicator of the risk of subsequent VF among CLHIV in our cohort on first‐line NNRTIs, given the low genetic barrier to resistance of these regimens. During the study follow‐up, CLHIV who had had at least one LLV episode after achieving virological suppression were classified as ever experiencing LLV. Those having more than one documented LLV event were classified as having multiple episodes of LLV. CLHIV who maintained virological suppression (pVL < 50 copies/mL) throughout the study period were classified as children sustaining virological suppression.

### 2.5 Study outcomes

Virological failure (VF) was defined as having a single pVL > 1000 copies/mL among CLHIV who had previously attained virological suppression on their first‐line cART. For CLHIV who had ever experienced LLV, VF must occur after their first episode of LLV. Loss to follow‐up was described as not presenting to the clinic for more than 12 months without documentation of transfer to another clinic or death.

### 2.6 Statistical analysis

Demographic and HIV‐related characteristics of CLHIV were summarized with number (n) and percentage (%) for categorical variables, and median and interquartile range (IQR) for continuous variables. The incidence rate of LLV was calculated by dividing the number of CLHIV with LLV by the total number of patient‐years of follow‐up (PYFU). The comparison of characteristics between children ever experiencing LLV versus children sustaining virological suppression were performed using Pearson’s Chi‐square or Fisher’s exact test, as appropriate, for categorical data, and Wilcoxon rank sum test for continuous data. Possible predictors of LLV were assessed using Poisson regression analysis. The associations between predictors and LLV were presented as incidence rate ratios (IRR).

In this study, baseline was defined as the visit date of the second pVL < 50 copies/mL for CLHIV with sustained virological suppression. For children ever experiencing LLV, the baseline was set at the time of the first LLV episode in order to prevent immortal time bias – the time during which the outcome of interest (e.g. VF) could not occur. All CLHIV were entered into the survival analysis at their baseline visit and were followed until having the study outcome (VF) or the end of study follow‐up. Children were censored at the time of the event or at the most recent visit for which a pVL was measured. The incidence rate of VF was calculated by dividing the number of CLHIV with VF by the total number of PYFU. Kaplan–Meier estimates and log‐rank test were performed to compare the cumulative probability of VF between those ever experiencing LLV versus sustaining virological suppression. Cox proportional hazards analysis was conducted to identify the predictors of VF. The associations between predictors and VF were summarized as hazard ratio (HR). Schoenfeld residuals were used to assess the statistical models for violations of the proportional hazards assumption and the models were accepted only after no violations were shown to occur.

Covariates for the Poisson and Cox proportional hazard models which demonstrated a *p* < 0.10 in univariable models were included in the multivariable models. Statistical significance was identified as a two‐sided *p* < 0.05. All statistical analyses were performed with SAS statistical software, version 9.4 (SAS Institute Inc, Cary, NC, USA) and Stata statistical software, version 14.1 (StataCorp LP, College, Station, TX, USA).

## 3 RESULTS

### 3.1 Characteristics of CLHIV

**Figure 1:**
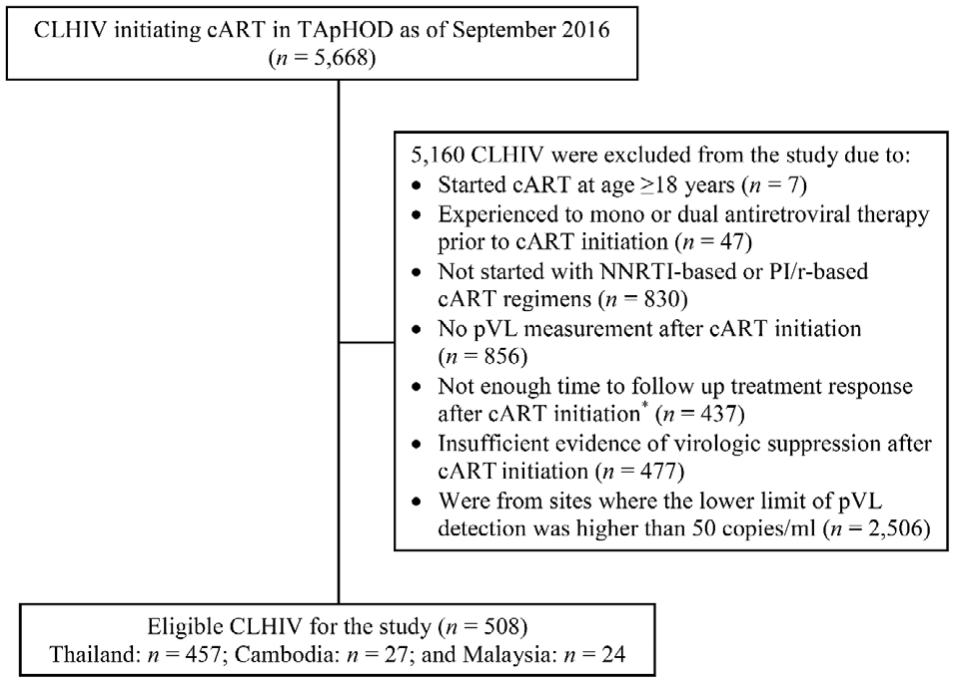
Flow diagram of study participants. cART, combination antiretroviral treatment; CLHIV, children living with HIV; NNRTI, non‐nucleoside reverse transcriptase inhibitor; PI/r, protease inhibitor‐boosted with ritonavir; pVL, plasma viral load; TApHOD, the TREAT Asia Pediatric HIV Observational Database. *Not enough time to follow up treatment response after cART initiation included 386 children who lost to follow‐up prior to a confirmation of virologic suppression, and 51 children who were on the first‐line cART regimen less than six months.

Of 5668 CLHIV in the cohort who had initiated cART as of September 2016, 508 were eligible for this study (Figure 1) and 55% were female. At cART initiation, the median age of CLHIV was 8.2 (IQR: 5.4 to 10.7) years, and 239 (47%) had advanced HIV clinical status (WHO clinical stage 3 and 4). About half of children (46%) initiated cART before 2005, whereas 28% initiated between 2005 and 2007, 18% between 2008 and 2011 and 8% between 2012 and 2016. The median nadir CD4 T‐cell percentage was 9% (IQR: 2% to 15%), and 63% had CD4 T‐cell percentage <15%. Among children with pVL performed at cART initiation (n = 328; 65%), the median peak pVL was 5.1 (IQR: 4.8 to 5.5) log_10_ copies/mL. At baseline, the median age was 9.6 (IQR: 7.0 to 12.3) years, WAZ was −1.3 (IQR: −1.9 to −0.4) and HAZ was −1.6 (IQR: −2.5 to −0.8). Almost all (97%) were on NNRTI‐based cART regimens, and the median duration of cART was 1.4 (IQR: 1.3 to 1.8) years. The median CD4 T‐cell percentage was 25% (IQR: 20 to 30%), and 51% had a CD4 T‐cell percentage ≥25%. The median frequency of pVL measurement was 1.2 (IQR: 0.8 to 1.5) time/year throughout the study period.

Over a median follow‐up time of 6.0 (IQR: 3.1 to 8.9) years from baseline, 86 CLHIV (17%) had ever experienced post‐suppression LLV, corresponding to an incidence rate of LLV of 2.8 (95% CI: 2.3 to 3.5) per 100 PYFU. Fifty‐four CLHIV (63%) had a single LLV episode, and 32 (37%) had multiple LLV episodes (16 with 2 episodes; 5 with 3 episodes; 7 with 4 episodes and 4 with ≥5 episodes). Of children with a single LLV episode (total = 54 episodes), 89% of the events were low magnitude LLV (50 to <400 copies/mL), and among children with multiple episodes of LLV (total = 108 episodes), 84% were low magnitude LLV. The comparison of demographic and HIV‐related characteristics at the time of cART initiation and at baseline between CLHIV ever experiencing LLV versus children with sustaining virological suppression are summarized in Table 1. Compared to CLHIV with sustained virological suppression, those who had ever experienced LLV during the study follow‐up were younger at baseline (8.1 vs. 9.8 years, *p* = 0.001), a higher proportion had pVL > 10 000 copies/mL at cART initiation (78% vs 56%, *p* < 0.001), and they had more frequent pVL measurements (1.3 vs. 1.1 times/year, *p* = 0.003). In the multivariable analysis, female sex (adjusted IRR (aIRR): 1.80; 95% confidence interval (95% CI): 1.29 to 2.52), living in Malaysia (aIRR: 6.85; 95% CI: 1.92 to 24.46, compared to Cambodia), having family members other than biological parents/grandparents as a primary caregiver (aIRR: 2.99; 95% CI: 1.95 to 4.58, compared to biological parents) and having baseline CD4 T‐cell percentage <25% (aIRR: 1.69; 95% CI: 1.22 to 2.35) were independently associated with an increased risk of LLV (Table 2).

**Table 1:** Characteristics of children living with HIV who were stable on first‐line combination antiretroviral treatment, stratified by low‐level viraemia experiences

Characteristics^a^	Ever experienced LLV (n = 86)	Sustained virological suppression (n = 422)	*p* ^b^
At cART initiation			
Age, years	6.7 (4.2 to 9.6)	8.4 (5.7 to 10.8)	0.004
Age category			0.09
<12 years	76 (88.4)	362 (85.8)	
12 to 14.9 years	7 (8.1)	56 (13.3)	
≥15 years	3 (3.5)	4 (0.9)	
Female sex	46 (53.5)	233 (55.2)	0.77
Country of residence			0.02
Thailand	74 (86.0)	383 (90.8)	
Malaysia	9 (10.5)	15 (3.5)	
Cambodia	3 (3.5)	24 (5.7)	
WHO clinical stage			0.96
Stage 1 to 2	33 (38.4)	170 (40.3)	
Stage 3	19 (22.1)	85 (20.1)	
Stage 4	24 (27.9)	111 (26.3)	
No event documented	10 (11.6)	56 (13.3)	
First‐line cART regimen			0.21
NNRTI‐based regimen	81 (94.2)	409 (96.9)	
NVP‐based	50 (58.1)	293 (69.4)	
EFV‐based	21 (24.5)	68 (16.1)	
Others	10 (11.6)	48 (11.4)	
PI/r‐based regimen	5 (5.8)	13 (3.1)	
LPV/r‐based	4 (4.7)	5 (1.2)	
Others	1 (1.1)	8 (1.9)	
Nadir CD4 T‐cell percentage, %	8 (2 to 15)	9 (2 to 15)	0.99
Nadir CD4 T‐cell percentagecategory			0.97
<15%	55 (64.0)	267 (63.3)	
≥15%	21 (24.4)	102 (24.2)	
Unknown	10 (11.6)	53 (13.5)	
Nadir CD4 T‐cell cell count^c^, cells/mm^3^	112 (39 to 334)	148 (30 to 320)	0.81
Nadir CD4 T‐cell count category^c^			0.90
<200 cells/mm^3^	37 (53.6)	195 (51.4)	
≥200 cells/mm^3^	25 (36.2)	139 (36.7)	
Unknown	7 (10.2)	45 (11.9)	
Peak pVL, log_10_ copies/mL	5.3 (4.9 to 5.6)	5 (4.8 to 5.5)	0.12
Peak pVL category			<0.001
<10,000 copies/mL	4 (4.7)	21 (5.0)	
≥10,000 copies/mL	67 (77.9)	236 (55.9)	
Unknown	15 (17.4)	165 (39.1)	
At baseline (At the 2nd pVL < 50 copies/mL)			
Age, years	8.1 (5.7 to 11.1)	9.8 (7.2 to 12.4)	0.001
Weight for age z‐score	−1.3 (−2.1 to −0.6)	−1.2 (−1.9 to −0.4)	0.21
Height for age z‐score	−1.6 (−2.5 to −0.8)	−1.6 (−2.5 to −0.8)	0.90
HIV status disclosed	7 (8.1)	71 (16.8)	0.44
Current cART regimen			0.21
NNRTI‐based	81 (94.2)	409 (96.9)	
PI/r‐based	5 (5.8)	13 (3.1)	
Period of cART initiation			0.09
<2005	47 (54.7)	184 (43.6)	
2005 to 2007	22 (25.6)	120 (28.4)	
2008 to 2011	15 (17.4)	78 (18.5)	
2012 to 2016	2 (2.3)	40 (9.5)	
Duration from cART initiation to baseline, years	1.4 (1.3 to 1.4)	1.4 (1.3 to 1.8)	0.008
CD4 T‐cell percentage, %	24 (20 to 31)	25 (20 to 30)	0.73
CD4 T‐cell percentage category			0.87
<25%	44 (51.2)	188 (44.5)	
≥25%	40 (46.2)	221 (52.4)	
Unknown	2 (2.3)	13 (3.1)	
CD4 T‐cell count^c^, cells/mm^3^	679 (505 to 889)	661 (480 to 886)	0.66
CD4 T‐cell count category^c^			0.83
<500 cells/mm^3^	16 (23.2)	101 (26.7)	
≥500 cells/mm^3^	51 (73.9)	267 (70.4)	
Unknown	2 (2.9)	11 (2.9)	
Frequency of pVL measurement, time/year	1.3 (1.0 to 1.5)	1.1 (0.8 to 1.5)	0.003

cART, combination antiretroviral therapy; EFV, efavirenz; LLV, low‐level viraemia; LPV/r, lopinavir/ritonavir; NVP, nevirapine, NNRTI, non‐nucleoside reverse transcriptase inhibitor; PI/r, protease inhibitor‐boosted with ritonavir; pVL, plasma viral load; WHO, World Health Organization.

^a^ Presented as n (%) for categorical data and median (interquartile range) for continuous data

^b^ The comparisons were performed using Pearson’s Chi‐square test or Fisher’s exact test, as appropriate, for categorical data, and Wilcoxon rank sum test for continuous data

^c^ Information on CD4 T‐cell count was collected for children aged ≥5 years (n = 448).

**Table 2:** Associated factors of low‐level viraemia among children living with HIV who were stable on first‐line combination antiretroviral treatment

Characteristics^a^	Univariable analysis^b^		Multivariable analysis^b^	
	Crude IRR (95% CI)	*p*	Adjusted IRR (95% CI)	*p*
Age		0.67		
<12 years	Reference			
12 to 14.9 years	0.84 (0.55 to 1.27)			
≥15 years	1.07 (0.52 to 2.18)			
Sex		0.001		<0.001
Male	Reference		Reference	
Female	1.56 (1.13 to 2.15)		1.80 (1.29 to 2.52)	
Country of residence		0.001		<0.001
Thailand	2.58 (0.82 to 8.09)		1.53 (0.47 to 4.91)	
Malaysia	7.04 (2.04 to 24.31)		6.85 (1.92 to 24.46)	
Cambodia	Reference		Reference	
Primary caregivers		<0.001		<0.001
One or both biological parents	Reference		Reference	
Grandparents	1.18 (0.76 to 1.84)		1.38 (0.87 to 2.20)	
Other family members	2.60 (1.75 to 3.88)		2.99 (1.95 to 4.58)	
Foster care	1.64 (0.59 to 4.59)		1.63 (0.57 to 4.62)	
Other non‐relatives	1.19 (0.65 to 2.15)		1.34 (0.72 to 2.48)	
Disclosure of HIV status		0.83		
Yes	0.95 (0.62 to 1.46)			
No	Reference			
Unknown	1.12 (0.74 to 1.69)			
Weight for age z‐score		0.01		0.05
< −2.5	Reference		Reference	
−2.5 to −1.5	0.42 (0.25 to 0.69)		0.48 (0.29 to 0.80)	
≥ −1.5	0.50 (0.31 to 0.80)		0.65 (0.40 to 1.05)	
Unknown	0.30 (0.07 to 1.26)		0.42 (0.09 to 1.83)	
Height for age z‐score		0.24		
< −2.5	Reference			
−2.5 to −1.5	0.76 (0.50 to 1.18)			
≥ −1.5	1.10 (0.76 to 1.59)			
Unknown	0.57 (0.14 to 2.35)			
WHO clinical stage at cART initiation		0.28		
Stage 1 to 2	Reference			
Stage 3	0.94 (0.61 to 1.43)			
Stage 4	0.95 (0.65 to 1.37)			
No event documented	0.59 (0.34 to 1.04)			
CD4 T‐cell percentage		0.008		0.001
< 25%	1.57 (1.15 to 2.15)		1.69 (1.22 to 2.35)	
≥ 25%	Reference		Reference	
Unknown	0.58 (0.14 to 2.36)		0.42 (0.10 to 1.78)	
Current cART regimen		0.03		0.08
NNRTI‐based	Reference		Reference	
PI/r‐based	2.35 (1.20 to 4.60)		2.04 (0.97 to 4.30)	
Period of cART initiation		0.58		
<2005	Reference			
2005 to 2007	0.80 (0.55 to 1.16)			
2008 to 2001	0.86 (0.53 to 1.39)			
2012 to 2016	0.70 (0.28 to 1.72)			

cART, combination antiretroviral therapy; CI, confidence interval; IRR, incidence rate ratio; NNRTI, non‐nucleoside reverse transcriptase inhibitor; PI/r, protease inhibitor‐boosted with ritonavir; WHO, World Health Organization.

^a^ Characteristics were evaluated at baseline, unless otherwise specified

^b^ The analyses were performed using Poisson regression models.

### 3.2 Incidence of virological failure

**Figure 2:**
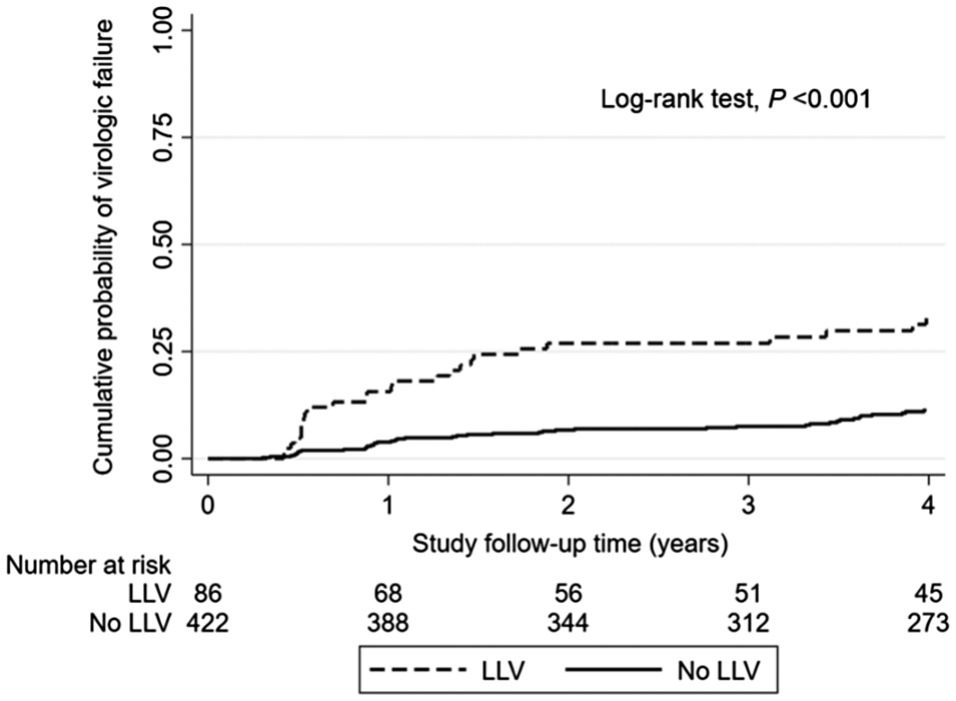
Kaplan–Meier estimates of unadjusted cumulative probability for virologic failure by low‐level viremia experienced during the first four years of study follow‐up. LLV, low‐level viremia.

Over the study follow‐up, 115 CLHIV (23%) developed VF after achieving virological suppression, corresponding to an overall incidence rate of 4.0 (95% CI: 3.4 to 4.9) events per 100 PYFU. VF was detected in 34 children (40%) who had experienced LLV, corresponding to an incidence rate of 8.9 (95% CI: 6.3 to 12.5) events per 100 PYFU; whereas VF was observed in 81 children (19%) who had previously sustained virological suppression, corresponding to an incidence rate of 3.3 (95% CI: 2.6 to 4.1) events per 100 PYFU (*p* log‐rank test <0.001). Kaplan–Meier estimates of unadjusted cumulative probability for VF by LLV experiences during the study follow‐up are illustrated in Figure 2. The overall median duration from baseline to the time of VF was 4.5 (IQR: 1.9 to 7.8) years, and the median duration from cART initiation to VF was 6.0 (IQR: 3.3 to 9.1) years.

In a sub‐analysis comparing the incidence of VF among CLHIV who had ever experienced high magnitude LLV (pVL 400 to 1000 copies/mL) and those who had low magnitude LLV (pVL 50 to <400 copies/mL), we noted that 13 out of 15 children with high magnitude LLV (87%) and 21 out of 71 children with low magnitude LLV (30%) developed a subsequent VF during the study follow‐up (incidence rate: 14.4 vs. 4.0 per 100 PYFU; *p* < 0.001). There were 30 CLHIV (6%) lost to follow‐up, of whom five ever experienced LLV and 25 had sustained virological suppression; there were no deaths during the study period.

### 3.3 Characteristics of children with virological failure

Of 115 CLHIV experiencing VF, 68 (59%) were female. Overall, at the time of VF, 95% were on NNRTI‐based cART regimens. The median age of CLHIV was 14.8 (IQR: 11.9 to 18.2) years, current CD4 T‐cell count was 469 (IQR: 317 to 624) cells/mm^3^ and current pVL was 4.1 (IQR: 3.4 to 4.7) log_10_ copies/mL. Among 34 CLHIV who had experienced LLV and subsequently developed VF, 21 (62%) had had a single LLV episode, whereas 13 (39%) had had multiple LLV episodes (6 with 2 episodes; 3 with 3 episodes; 1 with 4 episodes and 3 with ≥5 episodes) during the study period. The median duration from baseline to VF was 1.4 (IQR: 0.5 to 4.0) years, and the median duration from the last LLV to VF was 0.5 (IQR: 0.4 to 0.9) years. Comparing children who had ever experienced LLV with children who had previously sustained virological suppression, the clinical characteristics at the time of VF were not significantly different (*p *> 0.05), except for their current pVL (3.6 vs. 4.3 log_10_ copies/mL; *p* = 0.003) and the duration from baseline to VF (1.4 vs. 3.9 years; *p* = 0.002), which were higher among children in the latter group (Table 3).

**Table 3:** Characteristics of children living with HIV who were stable on first‐line combination antiretroviral treatment and had virological failure, stratified by low‐level viraemia experiences

Characteristics^a^, ^b^	Ever experienced LLV (n = 34)	Sustained virological suppression (n = 81)	*P* ^c^
Age, years	15.6 (12.6 to 18.3)	14.7 (11.8 to 17.5)	0.47
Female sex	21 (61.8)	47 (58.0)	0.71
WHO clinical stage at cART initiation			0.13
Stage 1 to 2	13 (38.3)	32 (39.5)	
Stage 3	8 (23.5)	16 (19.7)	
Stage 4	10 (29.4)	19 (23.5)	
No event documented	3 (8.8)	14 (17.3)	
Current CD4 T‐cell percentage, %	21 (14 to 26)	20 (13 to 26)	0.96
Current CD4 T‐cell count^d^, cells/mm^3^	463 (247 to 718)	474 (341 to 608)	0.95
pVL, log_10_ copies/mL	3.6 (3.2 to 4.3)	4.3 (3.6 to 4.8)	0.003
Current cART regimen			0.26
NNRTI‐based	31 (91.2)	78 (96.3)	
PI/r‐based	3 (8.8)	3 (3.7)	
Period of cART initiation			0.81
<2005	18 (52.9)	48 (59.3)	
2005 to 2007	9 (26.5)	15 (18.5)	
2008 to 2011	6 (17.7)	16 (19.8)	
2012 to 2016	1 (2.9)	2 (2.4)	
Duration from baseline to VF, years	1.4 (0.5 to 4.0)	3.9 (1.4 to 7.6)	0.002
Duration from cART initiation to VF, years	6.7 (4.4 to 9.7)	5.2 (2.9 to 9.1)	0.11

cART, combination antiretroviral therapy; LLV, low‐level viraemia; NNRTI, non‐nucleoside reverse transcriptase inhibitor; PI/r, protease inhibitor‐boosted with ritonavir; pVL, plasma viral load; VF, virological failure; WHO, World Health Organization.

^a^ Presented as n (%) for categorical data and median (interquartile range) for continuous data

^b^ Characteristics were evaluated at the time of virological failure, unless otherwise specified

^c^ The comparisons were performed using Pearson’s Chi‐square test or Fisher’s exact test, as appropriate, for categorical data, and Wilcoxon rank sum test for continuous data

^d^ Information on CD4 T‐cell count was collected for children aged ≥5 years (n = 100).

### 3.4 Predictors of virological failure

In the multivariable analysis, ever experiencing LLV during study follow‐up (adjusted hazard ratio (aHR): 3.01; 95% CI: 1.97 to 4.60), older age (age 12 to 14.9 years, aHR: 1.69; 95% CI: 1.08 to 2.66, age ≥15 years, aHR: 2.20; 95% CI: 1.02 to 4.76, compared with age <12 years) and baseline CD4 T‐cell percentage <25% (aHR: 1.90; 95% CI: 1.27 to 2.85) increased the risk of subsequent VF (Table 4). The proportional hazards assumption of the statistical model was tested and no evidence of violation was observed.

**Table 4:** Predictors of virological failure among children living with HIV who were stable on first‐line combination antiretroviral treatment

Characteristics^a^	Univariable analysis^b^		Multivariable analysis^b^	
	Crude HR (95% CI)	*p*	Adjusted HR (95% CI)	*p*
LLV experience		<0.001		<0.001
Sustained virologic suppression	Reference		Reference	
Ever experienced LLV	2.86 (1.90 to 4.28)		3.01 (1.97 to 4.60)	
Age		0.046		0.03
<12 years	Reference		Reference	
12 to 14.9 years	1.59 (1.04 to 2.48)		1.69 (1.08 to 2.66)	
≥15 years	2.02 (0.97 to 4.22)		2.20 (1.02 to 4.76)	
Sex		0.15		
Male	Reference			
Female	1.30 (0.90 to 1.92)			
Country of residence		0.003		0.07
Thailand	2.41 (0.59 to 8.87)		1.92 (0.46 to 8.07)	
Malaysia	8.04 (1.76 to 36.73)		4.47 (0.94 to 21.3)	
Cambodia	Reference		Reference	
WHO clinical stage at cART initiation		0.76		
Stage 1 to 2	Reference			
Stage 3	1.18 (0.72 to 1.94)			
Stage 4	0.90 (0.56 to 1.43)			
No event documented	0.90 (0.50 to 1.60)			
CD4 T‐cell percentage		0.001		0.002
<25%	1.84 (1.25 to 2.71)		1.90 (1.27 to 2.85)	
≥25%	Reference		Reference	
Unknown	3.50 (1.48 to 8.26)		2.82 (1.14 to 7.01)	
Current cART regimen		0.03		0.10
NNRTI‐based	Reference		Reference	
PI/r‐based	2.84 (1.24 to 6.52)		2.26 (0.94 to 5.44)	
Period of cART initiation		0.09		0.28
<2005	Reference		Reference	
2005 to 2007	0.94 (0.58 to 1.53)		1.13 (0.66 to 1.94)	
2008 to 2011	1.81 (1.08 to 3.04)		1.71 (0.97 to 3.03)	
2012 to 2016	0.79 (0.24 to 2.60)		0.83 (0.24 to 2.81)	

cART, combination antiretroviral therapy; CI, confidence interval; HR, hazard ratio; LLV, low‐level viraemia; NNRTI, non‐nucleoside reverse transcriptase inhibitor; PI/r, protease inhibitor‐boosted with ritonavir; WHO, World Health Organization.

^a^ Characteristics were evaluated at baseline, unless otherwise specified

^b^ The analyses were performed using Cox proportional hazards models.

## 4 DISCUSSION

In our cohort of Asian children with perinatally acquired HIV who were stable on first‐line suppressive cART regimens, approximately one‐fifth of children experienced at least one episode of LLV over the six‐year follow‐up period. LLV was predicted by sex, country of residence, primary caregiver and baseline immunological profiles. Importantly, we found that ever experiencing LLV during study follow‐up significantly increased the risk of subsequent VF. Therefore, LLV may be a reliable proxy for targeted pVL monitoring and can guide implementation of intensive adherence interventions in this population.

We noted that 17% of children in our cohort ever experienced at least one LLV episode following virological suppression after their first‐line cART initiation. Our prevalence of LLV is consistent with a previously published adult study in South Africa which noted that the prevalence of LLV (at least one pVL 51 to 999 copies/mL) was 23% among ALHIV receiving suppressive first‐line cART [25]. Comparison to other paediatric studies is challenging due to differing definitions of LLV used. In the UK and Ireland Collaborative HIV Paediatric Study (CHIPS), the prevalence of transient viraemia, which was defined as pVL ≥ 50 copies/mL followed by pVL < 50 copies/mL, was found in 22% of CLHIV on stable first‐ or second‐line suppressive cART regimens [8]. In addition, a study in Zimbabwe revealed that 23% of their CLHIV who had virological suppression after cART initiation developed transient LLV, which was defined as pVL ≥ 50 copies/mL followed by pVL < 50 copies/mL, over the study period [26]. Although one‐fifth of our CLHIV who did not have prior LLV developed VF at a rate of 3.3 events per 100 PYFU, VF was significantly higher among those who had previous LLV (*p* < 0.001). We also observed a short duration from the last LLV episode to VF in this study which might be attributed to an early pVL measurement in children experiencing LLV due to a high suspicion of subsequent VF.

The underlying mechanisms of LLV remain controversial. Plausible explanations include intermittent bursts of viral particles from latently infected reservoirs [27], low drug concentrations in blood because of suboptimal adherence or temporary alterations in pharmacokinetics [14], concurrent infections [12], intercurrent immunizations [28] or minor variations around the ranges of detection of commercial assays [29]. Other clinical factors have also been found to be linked to LLV, including advanced clinical stage of HIV prior to cART initiation, cART regimens and duration of viral suppression [30]. For this study, we noted that female sex, living in Malaysia (compared to Cambodia), having family members other than biological parents and grandparents as a primary caregiver, and low baseline CD4 T‐cell percentage were the predictors of LLV. However, we did not have data on self‐reported adherence or pill counts to assess their association with LLV in this population. The association between LLV and country of residence may be related to differences in the frequency of pVL testing across countries. In this study, Malaysian CLHIV had more frequent pVL measurements compared with Thai and Cambodian children (1.4 vs. 1.2 vs. 1.0 test/year respectively).

Our study demonstrated that ever experiencing LLV was linked to subsequent VF among treatment‐stable children on first‐line cART. This is supported by a study in a large multicentre rural‐urban South African cohort among adults receiving a suppressive first‐line cART, where LLV (pVL 51 to 999 copies/mL) significantly increased the risk of subsequent VF (pVL > 1000 copies/mL) (aHR: 2.6, 95% CI: 2.5 to 2.8) [25]. Additionally, in our sub‐analysis, we observed that the incidence of VF among CLHIV with high magnitude LLV (pVL 400 to 1000 copies/mL) was significantly greater when compared to children with low magnitude LLV (pVL 50 to <400 copies/mL) (*p* < 0.001). This finding suggests that high magnitude LLV may be useful as an indicator for subsequent VF risk in this population. Due to the non‐uniformity of LLV definitions used across paediatric studies, it is difficult to directly compare our findings. Nevertheless, the results from this study provide evidence that experiencing at least one LLV increases the risk of subsequent VF. This may help guide clinicians to more proactively implement interventions like intensive adherence monitoring and additional targeted pVL testing in children with LLV in order to prevent and detect early VF.

This study contains several limitations. Since we included only those sites in our network with pVL testing at a lower level of detection of <50 copies/mL to prevent chronological and misclassification biases, this led to the exclusion of a number of children with more limited pVL access and those with pVL testing using a less sensitive assay. This additionally reduced our ability to observe clear associations with single or multiple episodes of LLV and VF. We acknowledge that there are potential misclassification biases in this study [31]. First, defining VF as having single pVL > 1000 copies/mL could overestimate the incidence of VF. In addition, because all study sites routinely perform pVL testing at certain intervals following their national guidelines, we may have underestimated the incidence of LLV if it occurred during the interval between documented pVL tests, which could consequently attenuate the association between LLV and VF. Furthermore, LLV episodes could result in obtaining additional pVL testing, which would increase the potential to detect VF. A key missing variable was self‐ or clinic‐reported adherence, which would have been useful to assess as a risk factor for subsequent viraemia [14, 32]. Although this was not within the aims of the study, HIV drug resistance testing was not done among those with VF to determine whether LLV or VF at a given duration were driving mutation accumulation. As almost all of the CLHIV in our study were on NNRTI‐based regimens, our results cannot be generalized to those taking other HIV drug classes.

## 5 CONCLUSIONS

This study demonstrated the prevalence of LLV within our cohort, and how it increased the risk of subsequent VF among Asian CLHIV who were on stable first‐line suppressive cART. In resource‐limited settings, more intensive adherence interventions and potentially additional targeted pVL monitoring should be considered among children having LLV to prevent and detect early VF.

## COMPETING INTERESTS

AHS has received unrelated travel and project funding to her institution from ViiV Healthcare. Other authors declare no conflict of interest related to this study.

## AUTHORS’ CONTRIBUTIONS

TS developed the conception of research, designed the study, and developed the protocol. TS, KL, NK, MSF, RH, TB, LPS and RAN performed the data collection. ST, AK and TS designed and conducted the statistical analyses. TS contributed to the interpretation of study results. TS wrote the first draft and revised the manuscript. TS, AHS and VS provided critical revisions. All authors reviewed and approved the final version.

## ABBREVIATIONS

ALHIV, adults living with HIV; aHR, adjusted hazard ratio; aIRR, adjusted incidence rate ratio; cART, combination antiretroviral treatment; CHIPS, the UK and Ireland Collaborative HIV Paediatric Study; CLHIV, children living with HIV; HAZ, height for age z‐score; HR, hazard ratio; IeDEA, the International Epidemiology Databases to Evaluate AIDS; IQR, interquartile range; IRR, crude incidence rate ratio; LLV, low‐level viraemia; NNRTI, non‐nucleoside reverse transcriptase inhibitor; NRTI, nucleoside reverse transcriptase inhibitor; PI/r, protease inhibitor‐boosted with ritonavir; pVL, plasma viral load; PYFU, patient‐years of follow‐up; TApHOD, the TREAT Asia Pediatric HIV Observational Database; VF, virological failure; WAZ, weight for age z‐score; WHO, World Health Organization; 95% CI, 95% confidence interval.

## FUNDING

The TREAT Asia Pediatric HIV Observational Database is an initiative of TREAT Asia, a programme of amfAR, The Foundation for AIDS Research, with support from the U.S. National Institutes of Health’s National Institute of Allergy and Infectious Diseases, Eunice Kennedy Shriver National Institute of Child Health and Human Development, National Cancer Institute, National Institute of Mental Health, National Institute on Drug Abuse, the National Heart, Lung, and Blood Institute, the National Institute on Alcohol Abuse and Alcoholism, the National Institute of Diabetes and Digestive and Kidney Diseases, and the Fogarty International Center, as part of the International Epidemiology Databases to Evaluate AIDS (IeDEA; U01AI069907). The Kirby Institute is funded by the Australian Government Department of Health and Ageing, and is affiliated with the Faculty of Medicine, UNSW Australia.

## DISCLAIMER

The views expressed are those of the authors and does not necessarily represent the official views of any of the governments or institutions mentioned in the Funding Sources.
